# Metapath-guided topology and domain knowledge fusion for predicting miRNA–drug–disease triplet resistance and sensitivity associations

**DOI:** 10.1093/bib/bbag579

**Published:** 2026-11-07

**Authors:** Nan Sheng, Ling Gao, Xiongfeng Tang, Yunzhi Liu, Lan Huang, Yan Wang

**Affiliations:** Key laboratory of Symbol Computation and Knowledge Engineering of Ministry of Education, College of Computer Science and Technology, Jilin University, No. 2699 Qianjin Street, Changchun 130012, China; Key laboratory of Symbol Computation and Knowledge Engineering of Ministry of Education, College of Computer Science and Technology, Jilin University, No. 2699 Qianjin Street, Changchun 130012, China; The Orthopaedic Medical Center, Second Hospital of Jilin University, No. 218 Ziqiang Street, Changchun 130022, China; Key laboratory of Symbol Computation and Knowledge Engineering of Ministry of Education, College of Computer Science and Technology, Jilin University, No. 2699 Qianjin Street, Changchun 130012, China; Key laboratory of Symbol Computation and Knowledge Engineering of Ministry of Education, College of Computer Science and Technology, Jilin University, No. 2699 Qianjin Street, Changchun 130012, China; Key laboratory of Symbol Computation and Knowledge Engineering of Ministry of Education, College of Computer Science and Technology, Jilin University, No. 2699 Qianjin Street, Changchun 130012, China

**Keywords:** miRNA–drug–disease association, drug resistance and sensitivity, heterogeneous graph, metapath, domain knowledge

## Abstract

Accurately predicting drug resistance and sensitivity is critical for understanding disease treatment mechanisms and improving therapeutic strategies. MicroRNAs (miRNAs) play essential roles in regulating drug response. However, existing computational methods mainly focus on miRNA–drug pairwise associations and overlook the disease-specific context. In this study, we propose a metapath-guided topology and domain knowledge fusion framework (MTDKF) for predicting miRNA–drug–disease triplet resistance (MRDR) and sensitivity (MRDS) associations. Specifically, we construct a triplet heterogeneous graph to model the interactions among miRNAs, drugs, and diseases. A metapath topology view encoder is designed to capture regulatory patterns between nodes. Additionally, a domain knowledge view encoder is introduced to incorporate sequence, structural, and semantic features of biological entities. These two views are further integrated to learn robust and discriminative representation. We manually curate two benchmark datasets from experimentally validated associations. Extensive experimental results demonstrate that MTDKF consistently outperforms state-of-the-art approaches across multiple evaluation metrics. Furthermore, case studies on two breast cancer drugs doxorubicin and tamoxifen demonstrate its potential in discovering novel miRNAs related to drug resistance and sensitivity under disease-specific contexts.

## Introduction

Diseases have long posed a major threat to human health and life, among which cancer represents a substantial global burden and remains one of the leading causes of death worldwide [1]. Chemotherapy continues to serve as a primary and conventional treatment for many cancer types. However, the emergence of drug resistance during chemotherapy represents a critical barrier to achieving long-term remission and eventual cure; this resistance may arise from both acquired and intrinsic mechanisms [2]. The microRNAs (miRNAs) are a class of small noncoding RNAs, typically 19–25 nucleotides in length, that regulate gene expression by targeting specific genes or modulating processes such as cell proliferation, cell cycle progression, and apoptosis, thereby influencing cellular responses to chemotherapeutic agents and even enhancing drug sensitivity [3]. For instance, Wang *et al*. [4] reported that miR-369-3p reverses resistance to osimertinib in lung cancer cells by targeting the protein PTPN12. Similarly, Shi *et al*. [5] demonstrated that miR-142-3p enhances paclitaxel sensitivity in resistant breast cancer by targeting GNB2. In addition, the knockdown of miR-3689a-3p significantly increases resistance to sorafenib, whereas its overexpression improves the therapeutic response in hepatocellular carcinoma [6]. These findings highlight the critical role of miRNAs in modulating drug resistance and sensitivity, underscoring the importance of systematically exploring miRNA-mediated drug response mechanisms for rational drug development and optimized clinical treatment strategies.

Traditional experimental approaches, such as wet-lab assays, are often time-consuming, costly, and subject to experimental variability, making them unsuitable for large-scale screening of potential resistance and sensitivity associations. With the rapid accumulation of biological data, computational methods have emerged as promising alternatives for efficiently identifying candidate miRNA–drug associations. In recent years, several computational approaches have been proposed to predict miRNA-mediated drug resistance and sensitivity. For example, Huang *et al*. [7] employed graph convolutional networks on miRNA–drug bipartite graph to identify drug resistance-related miRNAs. Deng *et al*. [8] developed a dual-channel heterogeneous graph neural network to extract latent node features from miRNA–drug bipartite graph and predict drug sensitivity-related miRNAs. Wei *et al*. [9] considered miRNA–drug bipartite graph sparsity using collaborative filtering and contrastive learning techniques. More recently, Xie *et al*. [10] integrated molecular language models as node features to predict ncRNA–drug associations. In contrast, Sheng *et al*. [11–13] proposed several deep learning models incorporating biological attributes, including DLST-MDA, MGCNA, and PLMF-MDA. DLST-MDA combines convolutional neural networks and graph convolutional networks to learn representations from miRNA sequences and drug structures. MGCNA integrates gene regulatory information through multi-view graph convolutional networks, while PLMF-MDA leverages pretrained language models to infer drug resistance and sensitivity-related miRNAs. Furthermore, Zhang *et al*. [14] integrated sequence-based pretrained features, structural information, and biological attributes to improve prediction performance.

Despite their promising results, existing methods primarily focus on miRNA–drug pairwise associations, neglecting the critical role of disease context. In real-world clinical settings, the regulatory effects of miRNAs on drug response are highly disease-specific. The same miRNA–drug pairwise may exhibit markedly different, or even opposite, effects across different diseases or pathological conditions. As shown in Fig. 1, the miRNA hsa-let-7a-5p induces resistance to doxorubicin in prostate cancer and hepatocellular carcinoma [15, 16], while enhancing sensitivity in breast cancer [17]. Therefore, modeling miRNA–drug interactions without incorporating disease context is insufficient to capture the underlying biological complexity of drug responses.

**Figure 1:**
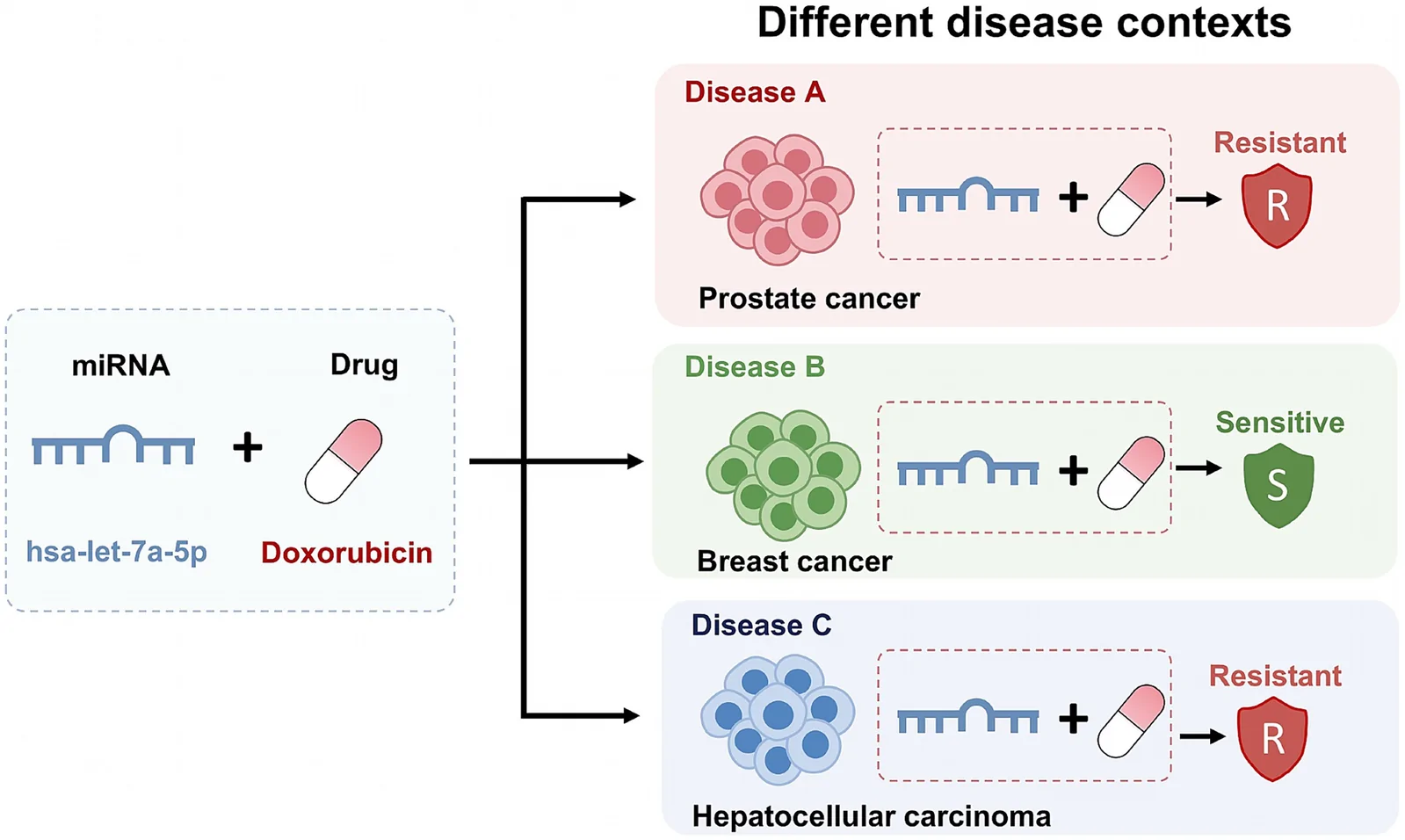
An example of hsa-let-7a-5-doxorubicin pairwise produces different drug responses in different disease contexts.

In this paper, we study miRNA–drug–disease triplet relationships and propose a metapath-guided topology and domain knowledge fusion framework (MTDKF) for predicting miRNA–drug–disease triplet resistance (MRDR) and sensitivity (MRDS) associations. Specifically, we design a dual-view learning strategy that integrates a metapath topology view encoder and a domain knowledge view encoder to capture both node topology and intrinsic features. In the metapath topology view, the node embedding is learned by propagating information along multiple predefined metapaths to model topological relationships. In the domain knowledge view, the node embedding is derived from biological attributes of each entity, capturing complementary intrinsic feature. The representations from both views are then fused to enhance prediction performance. The main contributions of this study are as follows:

(1) To the best of our knowledge, this is the first study to model MRDR and MRDS association prediction. Unlike existing pairwise approaches, MTDKF explicitly incorporates disease-specific context, enabling more biologically realistic modeling of drug response mechanisms.
(2) We propose a dual-view learning framework that combines a metapath topology encoder for capturing regulatory patterns and a domain knowledge encoder for learning intrinsic biological attributes, thereby enhancing representation learning.
(3) We manually constructed two benchmark datasets, MRDR-dataset and MRDS-dataset, and the experimental results show that MTDKF consistently outperforms state-of-the-art methods. Further case studies validate its ability to identify novel miRNAs associated with drug resistance and sensitivity.

## Materials and methods

### Benchmark datasets

This study focuses on modeling MRDR and MRDS associations by integrating triplet relationships and corresponding biological attributes. It consists of two types of data: (i) **Association data.** Due to the lack of publicly available miRNA–drug–disease (MRD) triplet datasets, we manually constructed two benchmark datasets, namely MRDR-dataset and MRDS-dataset, based on the latest version of the ncRNADrug database [18]. This database provides curated and predicted associations between noncoding RNAs (including miRNAs, long noncoding RNAs, and circular RNAs) and drug resistance/sensitivity. In this work, we only retain experimentally validated human MRD triple associations. To ensure consistency across different data sources, the three types of biological entities are further standardized. MiRNA names are normalized according to miRBase [19], and mature miRNA identifiers are used as the canonical form. Drug names and synonyms are mapped to their corresponding DrugBank [20], entries. Disease names and synonymous terms are normalized according to the Disease Ontology (DO) [21]. After preprocessing and filtering, the MRDR-dataset contains 6494 resistance triplets involving 1316 miRNAs, 104 drugs, and 81 diseases, while the MRDS-dataset includes 6599 sensitivity triplets with 1247 miRNAs, 142 drugs, and 92 diseases. (ii) **Attribute data.** For node domain knowledge construction, miRNA sequences and drug SMILES are obtained from miRBase [19] and DrugBank [20], respectively. Disease terms are standardized using the DO dataset [21]. The statistical details of the datasets are summarized in Table 1.

**Table 1:** The statistics of the manually curated MRDR and MRDS benchmark datasets.

	miRNAs	Drugs	Diseases	Associations
MRDR-dataset	1316	104	81	6494
MRDS-dataset	1247	142	92	6599

### Overview

The overall architecture of MTDKF is illustrated in Fig. 2. The proposed model consists of three major components: (i) a metapath topology view encoder, (ii) a domain knowledge view encoder, and (iii) a multi-view representation fusion and prediction module. First, the metapath topology view encoder is designed to capture regulatory relationships among miRNAs, drugs, and diseases from the constructed MRD heterogeneous graph by leveraging predefined metapaths. Second, the domain knowledge view encoder aims to extract complementary attribute-level information beyond graph topology by incorporating miRNA sequence features, drug molecular structure information, and disease semantic representations. Third, a weighted fusion mechanism is employed to integrate the learned representations from the two views, resulting in robust and information node embeddings. Finally, the concatenated embeddings of miRNA, drug, and disease nodes are fed into a multilayer perceptron (MLP) to predict the probability of resistance or sensitivity associations for each MRD triplet.

**Figure 2:**
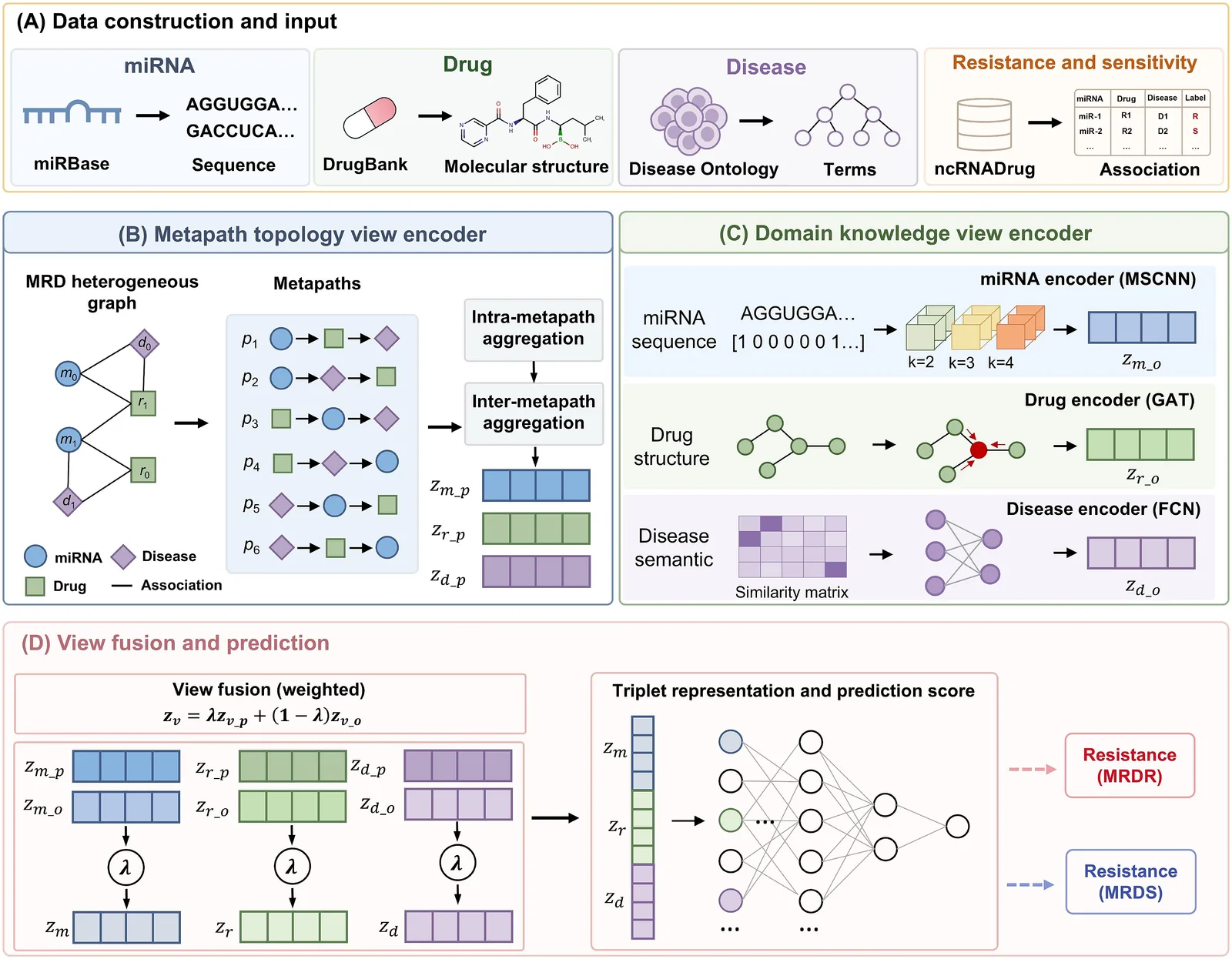
Overview of the proposed MTDKF framework.

### Problem formulation

Let $M = \{m_{1}, m_{2}, \dots , m_{N_{m}}\}$, $R = \{r_{1}, r_{2}, \dots , r_{N_{r}}\}$, and $D = \{d_{1}, d_{2}, \dots , d_{N_{d}}\}$ denote the sets of miRNAs, drugs, and diseases, respectively. The Cartesian product $S=M \times R \times D$ represents all possible MRD triplets. A triplet *(m,r,d)∈ S* is labeled as positive *y=1* if the triple entities are known to be associated, and negative *y=0* otherwise, representing unknown or unobserved associations. The goal of this study is to learn an effective model to identify potential MRDR and MRDS from these unknown association.

### Metapath topology view encoder

#### Construction of MRD heterogeneous graph

To capture the complex interactions underlying drug resistance and sensitivity, we construct an MRD heterogeneous graph *G=(V,E)*, where $V=\left \{M \cup R \cup D \right \}$ denotes the set of nodes and *E* represents the set of pairwise associations among them. Each node is initialized with biologically meaningful features. For miRNAs, we adopt k-mer frequency descriptors to encode sequence information. Given the short length of miRNA sequences, 1-mer, 2-mer, and 3-mer feature are combined to generate an 84D feature vector, resulting in the feature matrix $X_{m} \in \mathbb{R}^{N_{m} \times f_{m}}$. For drugs, MACCS fingerprints are used to construct $X_{r}\in R^{N_{r} \times f_{r}}$, while disease features $X_{d} \in \mathbb{R}^{N_{d} \times f_{d}}$ are derived from semantic similarity based on Disease Ontology. Here, $N_{m}$, $N_{r}$, and $N_{d}$ represent the number of miRNAs, drugs, and diseases respectively, while $f_{m}$, $f_{r}$, and $f_{d}$ denote the feature dimensions of miRNAs, drugs, and diseases, respectively.

#### Node feature transformation

Since MRD heterogeneous graphs contain different types of biological entity nodes, their features are obtained through different computational methods and have unequal feature vector dimensions, resulting in these features residing in different spaces. So first, we need to project the features of miRNAs, drugs, and disease nodes into a unified embedding space. Specifically, for a node *v* of type $\phi \in \{M, R, D\}$, a type-specific linear transformation is applied:


(1) \begin{align*}& h_{v}=\sigma\left(W_\phi \cdot x_{v}+b_\phi\right),\end{align*}

where $W_\phi $ and $b_\phi $ are learnable weight parameters, $x_{v}$ is the input feature, and $h_{v}$ is the transformed representation of node *v*. $\sigma (\cdot )$ is an activation function.

#### Metapath generation and message aggregation

**Metapath generation.** Metapaths provide an effective means of capturing regulatory mechanisms among miRNAs, drugs, and diseases. Here, to model potential interactions among miRNAs (M), drugs (R), and diseases (D), we define six metapaths: M-R-D, M-D-R, R-M-D, R-D-M, D-M-R, and D-R-M. Each metapath describes a specific information flow pattern. For example, M-R-D indicates that an miRNA may influence disease response through drug regulation. For each metapath *p∈ P*, a corresponding directed subgraph $G_{p}=(V,E_{p})$ is constructed from the heterogeneous graph *G*. $E_{p}$ denotes the set of edges defined by the metapath, and each subgraph contains corresponding metapath instances.

**Intra-metapath aggregation.** Given a metapath instance $s\in S_{p}$ within a subgraph, it can be represented as a relational path $h \xrightarrow{r_{he}} e \xrightarrow{r_{et}} t$, where *h*, *e*, and *t* denote the head, intermediate, and tail node, respectively, and $r_{he}$ and $r_{et}$ represent the corresponding relations. This formulation ensures that information is propagated from the head node to the tail node via the intermediate node. For each metapath instance embedding $z_{s}^{p}$, we adopt the approach proposed by [22, 23] to model the relationships among nodes, which is defined as


(2) \begin{align*}& z_{s}^{p}=\frac{1}{3}\left(\left(h_{h} \odot r_{he}+h_{e}\right) \odot r_{et}+h_{t}\right),\end{align*}

where $h_{h}$, $h_{e}$, and $h_{t}$ are node embeddings, $r_{he}$, $r_{et}$ are relation embeddings, and *⊙ * denotes element-wise multiplication. After encoding metapath instances into vector representations, we employ a graph attention layer [24] to aggregate semantic information by treating all metapath instances associated with a target node *v∈ V* as its neighbors. The key idea is that different metapath instances contribute unequally to the representation of the target node.


(3) \begin{align*} & m_{v}^{p}=\operatorname{LeakyReLU}\left(\boldsymbol{a}_{p}^{T} \cdot\left[h_{v} \| z_{s}^{p}\right]\right)\end{align*}

(4) \begin{align*} & \alpha_{sv}^{p}=\frac{\exp \left(m_{v}^{p}\right)}{\sum_{s^{\prime} \in S_{p}(v)} \exp \left(m_{s^{\prime}}^{p}\right)}\end{align*}

(5) \begin{align*} & h_{v}^{p}=\sigma\left(\sum_{s \in S_{p}(v)} \alpha_{s v}^{p} \cdot z_{s}^{p}\right), \end{align*}

where $\boldsymbol{a}_{p}^{T}$ denotes the parameterized attention vector specific to metapath *p*, and ∥ represents the vector concatenation operator. The $m_{v}^{p}$ measures the importance of a metapath instance *s* with respect to node *v*, while $\alpha _{sv}^{p}$ denotes the normalized importance weight of each metapath instance. The function $\sigma (\cdot )$ represents a nonlinear activation function. To further stabilize the learning process and mitigate the high variance introduced by structural heterogeneity in the graph, we incorporate a multi-head attention mechanism. The outputs of multiple attention heads are then concatenated to form the final representation.

**Inter-metapath aggregation.** After aggregating node representations from the six subgraphs, we further employ an inter-metapath aggregation layer to integrate the semantic information encoded by all metapaths (subgraphs). Considering that different metapaths are not equally important in the MRD heterogeneous graph, we introduce an attention mechanism to assign adaptive weights to different metapaths.


(6) \begin{align*} & e_\phi^{p}=\frac{1}{|\phi|} \sum_{v \in \phi} q_\phi^{T} \cdot \tanh \left(W_{\varphi} \cdot h_{v}^{p}+b_{\varphi}\right)\end{align*}

(7) \begin{align*} & \beta_\phi^{p}=\frac{\exp \left(e_\phi^{p}\right)}{\sum_{p^{\prime} \in P} \exp \left(e_\phi^{p^{\prime}}\right)}\end{align*}

(8) \begin{align*} & z_{v}=\sum_{p \in P} \beta_\phi^{p} \cdot h_{v}^{p}, \end{align*}

where $W_{\varphi }$ and $b_{\varphi }$ are learnable parameters, and $q_\phi ^{T}$ denotes the semantic-level attention vector. The coefficient $\beta _\phi ^{p}$ can be interpreted as the relative importance of metapath *p* for nodes of type *ϕ *. The $z_{v}$ denotes the final metapath-enhanced representation of node *v* after inter-metapath aggregation. Accordingly, the resulting representations of miRNA, drug, and diseaseare denoted as $z_{m{\_}p}\in \mathbb{R}^{1\times d}$, $z_{r{\_}p}\in \mathbb{R}^{1\times d}$, and $z_{d{\_}p}\in \mathbb{R}^{1\times d}$, respectively.

### Domain knowledge view encoder

#### miRNA sequence knowledge encoder

To capture functional-related domain knowledge features from miRNA nucleotide sequences, we employ a multi-scale convolutional neural network (MSCNN) to encode sequence-level features. Given a miRNA sequence $m_{i}$, each nucleotide $b_{j}\in \{A,U,G,C\}$ is first transformed into a one-hot representation, resulting in an *L× 4* matrix, where *L* denotes the sequence length. In our datasets, 98% of miRNA sequences are <24 nucleotides in length (Supplementary Figure S1). Therefore, all sequences are normalized to a fixed length of 24 via truncation or zero-padding. The one-hot representation is then mapped to a dense embedding matrix $X_{i}^{seq}$ through a learnable embedding layer. To capture sequence patterns at different scales, we adopt *K* parallel convolutional branches with different kernel sizes. Each branch consists of a 1D convolution layer followed by a nonlinear activation and max-pooling operation:


(9) \begin{align*}& F_{i}^{(k)}=\operatorname{MaxPool}\left(\sigma\left(\operatorname{Conv1D}{} _{k}\left(X_{i}^{\textrm{seq }}\right)\right)\right)\end{align*}

The resulting feature maps from all branches are concatenated to form a multi-scale representation, which is further transformed by a fully connected layer to obtain the final miRNA sequence embedding $z_{m{\_}o}\in \mathbb{R}^{1\times d}$.

#### Drug structural knowledge encoder

This module employs a graph attention network (GAT) [24] to learn representations of drug molecular graphs, aiming to encode structural domain knowledge of drugs. Each drug as a molecular graph $G_{r}=(V_{r},E_{r})$, where nodes correspond to atoms and edges represent chemical bonds. Each atom is initialized with a feature vector encoding its chemical properties, such as atom type, degree, hybridization state, and formal charge. Given $G_{r}$, a multilayer GAT is applied to iteratively update atom representations. Specifically, the representation $h_{i}^{(l)}$ of atom *i* at the *l*th layer is updated as follows:


(10) \begin{align*} & e_{i j}^{(l)}=\operatorname{LeakyReLU}\left(w^{(l)^{T}}\left[W^{(l)} h_{i}^{(l-1)} \| W^{(l)} h_{j}^{(l-1)}\right]\right.\end{align*}

(11) \begin{align*} & a_{ij}^{(l)}=\frac{\exp \left(e_{i j}^{(l)}\right)}{\left.\sum_{k \in \mathcal{N}_{i}} \exp \left(e_{i k}^{(l)}\right)\right)}\end{align*}

(12) \begin{align*} & h_{i}^{(l)}=\sigma\left(\sum_{j \in \mathcal{N}_{i}} a_{i j}^{(l)} \cdot W^{(l)} h_{j}^{(l-1)}\right), \end{align*}

where $w^{(l)}$ and $W^{(l)}$ are learnable parameter vector and matrix, respectively. ∥ denotes the vector concatenation operation; and $\sigma (\cdot )$ represents a nonlinear activation function. $\mathcal{N}_{i}$ denotes the set of neighboring nodes of atom *i*. Here, multi-head attention is employed to capture diverse local chemical semantics in parallel. To obtain a graph-level representation, we apply a readout function combining global mean pooling and max pooling over all atoms. The resulting vector is used as the final drug embedding $z_{r{\_}o}\in \mathbb{R}^{1\times d}$.

#### Disease semantic knowledge encoder

Here, we utilize disease semantic information to characterize the biomedical features of disease nodes. The DO database provides a standardized vocabulary for human diseases, offering consistent descriptions of disease terms and associated phenotypic characteristics. In the DO database, disease ontology terms are organized in a hierarchical tree structure [21]. Following prior work [25], we compute disease semantic similarity matrix $D^{sem} \in \mathbb{R}^{N_{d}\times N_{d}}$ based on ontology structure. The similarity vectors are then projected into a low-dimensional embedding space using a multilayer fully connected network:


(13) \begin{align*}& Z_{d}^{(l)}={ReLU}\left(W_{d}^{(l)} \cdot Z_{d}^{(l-1)}+b_{d}^{(l)}\right),\end{align*}

where $W_{d}^{(l)}$ and $b_{d}^{(l)}$ denote the learnable weight matrix and bias vector at the *l*th layer, respectively, and $Z_{d}^{(l)}$ represents the disease embedding at layer *l*, with $Z_{d}^{(0)}=D^{sem}$. The final disease representation is denoted as $z_{d{\_}o}\in \mathbb{R}^{1\times d}$.

### View fusion and model optimization

Through the multi-view encoding modules introduced above, we obtain metapath-based representations $z_{m{\_}p}$, $z_{r{\_}p}$, and $z_{d{\_}p}$, as well as domain knowledge-based representations $z_{m{\_}o}$, $z_{r{\_}o}$, and $z_{d{\_}o}$ for miRNAs, drugs, and diseases, respectively. These representations are integrated via a weighted fusion mechanism:


(14) \begin{align*}& z_{v}=\lambda z_{v{\_}p}+(1-\lambda) z_{v{\_}o}, v \in \mathcal{V}=\{M \cup R \cup D\},\end{align*}

where $\lambda \in [0, 1]$ controls the contribution of each view. For a given MRD triplet *(m,r,d)*, the corresponding node embeddings are concatenated and fed into the MLP to predict the association score:


(15) \begin{align*}& \hat{y}={Sigmoid}\left({MLP}\left(z_{m}\left\|z_{r}\right\| z_{d}\right)\right)\end{align*}

Finally, the proposed model is optimized using the loss function adopted in [26], defined as


(16) \begin{align*}& \mathcal{L}=(1-\alpha)\|y \odot(y-\hat{y})\|^{2}+\alpha\|(1-y) \odot(y-\hat{y})\|^{2},\end{align*}

where *α * is a balancing coefficient, $\|\cdot \|^{2}$ denotes the $L_{2}$-norm, and *y* and $\hat{y}$ represent the ground-truth labels and predicted scores of the input triplets, respectively.

## Experiments and results

### Experimental setup

**Evaluation protocols.** We randomly split the dataset into 90% for cross-validation (cv) and 10% for independent testing. Five-fold cv strategy is adopted for model training and optimize the hyper-parameters, while the independent test set is performed for final evaluation. All experimentally validated MRDR and MRDS associations are treated as positive samples. Negative samples are generated by randomly combining miRNAs, drugs, and diseases without known associations. In each cv iteration, we randomly select four out of five positive samples in the cv set and generate an equal number of negative samples as the training set. To prevent information leakage, the heterogeneous graph is reconstructed independently for each fold using only the positive associations in the corresponding training set. Associations from the validation fold are excluded from graph construction and are used solely for evaluation. Similarly, when performing the independent test, associations in the independent test set are not incorporated into the heterogeneous graph. During validation and testing, 30 corresponding negative samples are generated for each test triplet, so as to sort the prediction scores of all samples. To assess the MTDKF’s ability, we focus on three evaluation metrics commonly used in recommender systems, covering hit ratio (Hit@n) (*n=1,3,5*), normalized discounted cumulative gain (NDCG@n) (*n=1,3,5*), and Mean Reciprocal Rank (MRR).

**Implementation details.** The proposed model MTDKF is implemented using PyTorch and optimized with the Adam optimizer (learning rate=0.001). Early stopping is applied with a patience of 50 to terminate the training early. The kernel sizes of the MSCNN are set to 2, 3, and 4, and the number of GAT layers for drug encoding is set to 3. The fusion coefficient *λ * and loss balance coefficient *α * are both set to 0.4. To maintain a consistent experimental setting and avoid dataset-specific over-optimization, hyperparameter analysis is performed on the MRDR-dataset, and the resulting configuration is applied unchanged to the MRDS-dataset. Additional parameters and implementation details are available at GitHub: https://github.com/sheng-n/MTDKF.

### Comparison with existing methods on benchmark datasets

To evaluate the effectiveness of the proposed method MTDKF, we compare it with several representative approaches, including machine learning methods (Random Forest and XGBoost) and graph-based models (GCLMTP [27], CSGLMD [28], MCHNN [29], and HCMGNN [23]). A brief description of baseline methods is provided in the supplementary file. For Random Forest and XGBoost, we use the attribute features of the miRNA, drug, and disease nodes as input. The experimental results of MTDKF and baseline methods in five-fold cv and independent test for MRDR-dataset and MRDS-dataset datasets are summarized in Fig. 3 and Supplementary Table S1.

**Figure 3:**
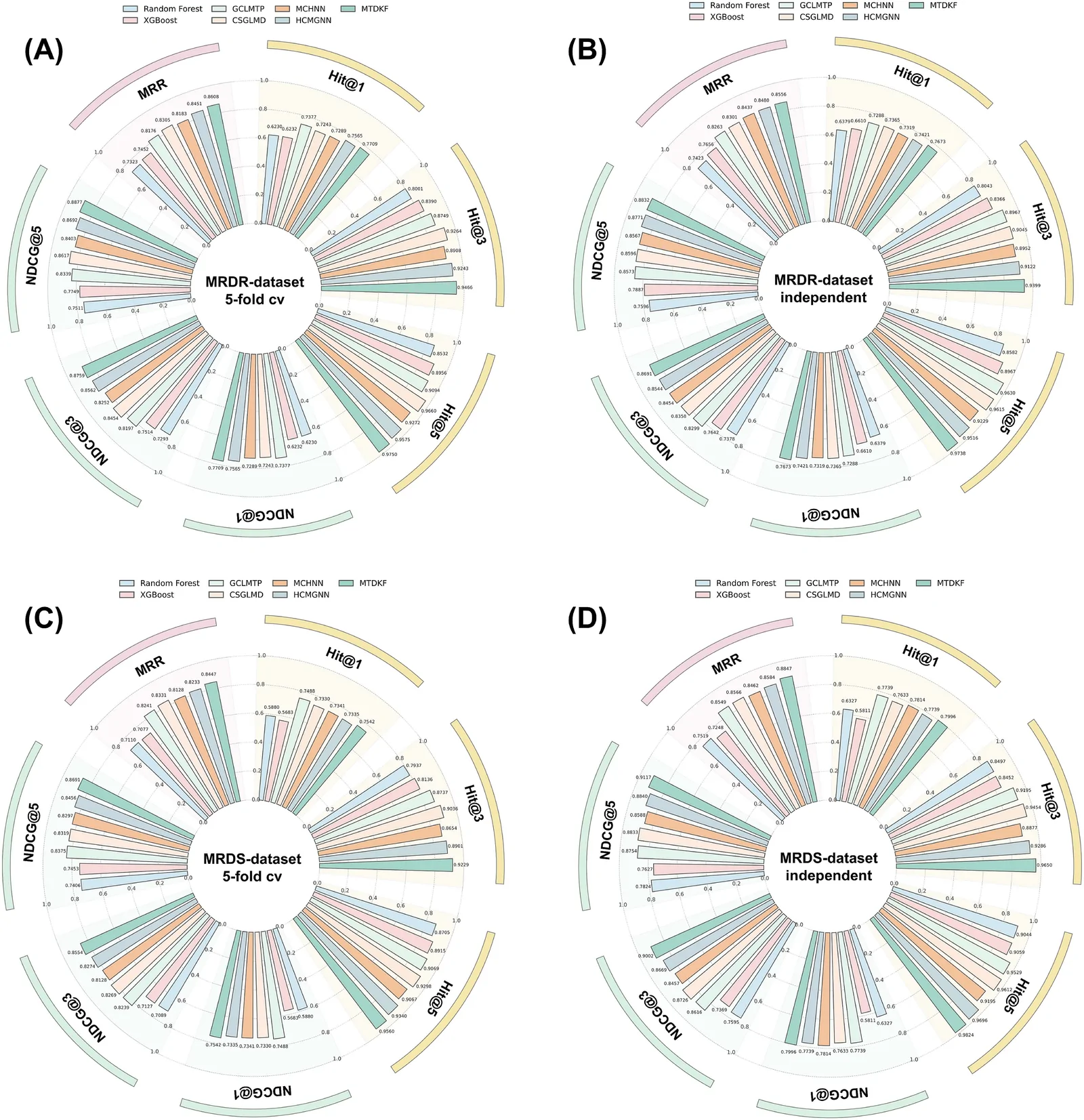
Performance comparison between MTDKF and baseline methods on the MRDR-dataset and MRDS-dataset under five-fold cv and independent test set.

From the results, several observations can be made. (i) MTDKF consistently outperforms other baseline methods and achieves the most competitive performance across both datasets, demonstrating its effectiveness in predicting MRDR and MRDS associations. (ii) Compared with Random Forest and XGBoost, which rely solely on node attribute features, MTDKF shows substantial performance improvements, indicating that incorporating association information is essential for capturing complex MRD interactions. (iii) Graph-based models GCLMTP, CSGLMD, and MCHNN also achieve strong performance, suggesting that topological information plays an important role in improving prediction accuracy. (iv) HCMGNN achieves performance on two datasets by modeling triplet relationships via meta-paths, highlighting the advantage of leveraging higher-order structural information. Overall, MTDKF demonstrates consistent superiority across different datasets and evaluation settings. This improvement can be attributed to its ability to jointly exploit topology information and domain knowledge, enabling complementary information integration for more accurate MRD association prediction.

### Variant analysis

To assess the contribution of each component, we design two variants of MTDKF: MTDKF (w/o MT) and MTDKF (w/o DK). The former removes the metapath topology view and relies on domain knowledge features, whereas the latter excludes domain knowledge view and utilizes metapath information. The results on the independent test sets of MRDR-dataset and MRDS-dataset are shown in Fig. 4. From Fig. 4, we have the following observations: (i) The results of MTDKF consistently outperformed the two variants across the two datasets, demonstrating the effectiveness and necessity of integrating metapath topology and domain knowledge information. (ii) MTDKF (w/o MT) shows a substantial performance degradation, indicating that relying solely on domain knowledge is insufficient to fully characterize node feature. (iii) MTDKF (w/o DK) also degrades performance, suggesting that biological attributes provide complementary information beyond graph structure.

**Figure 4:**
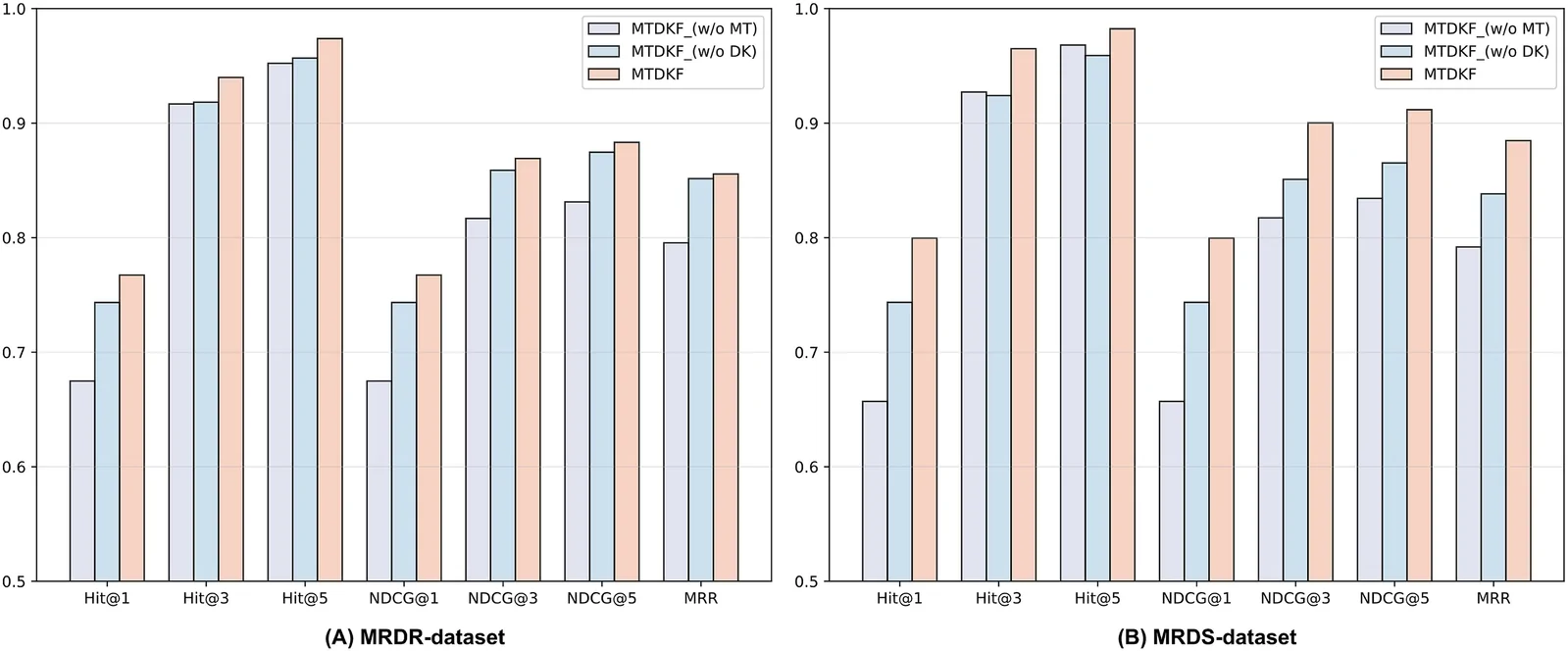
Performances of MTDKF and its variants in ablation study.

### Analysis of hyper-parameters

In this section, we analyze the sensitivity of three key hyper-parameters, including the fusion coefficient *λ *, the loss balance coefficient *α *, and the embedding dimension *d*. All experiments are conducted on the MRDR-dataset using five-fold cv.

**Analysis of fusion coefficient *λ *:** The fusion coefficient *λ * controls the relative contribution of the metapath topology view and the domain knowledge view. As shown in Fig. 5A, the model performance first improves and then declines as *λ * increases. The best performance is achieved at *λ =0.4*, indicating that a balanced integration of structural and domain knowledge features yields the most effective representations. Larger values of *λ * lead to performance degradation, suggesting that overemphasizing structural information weakens the contribution of domain knowledge.

**Figure 5:**
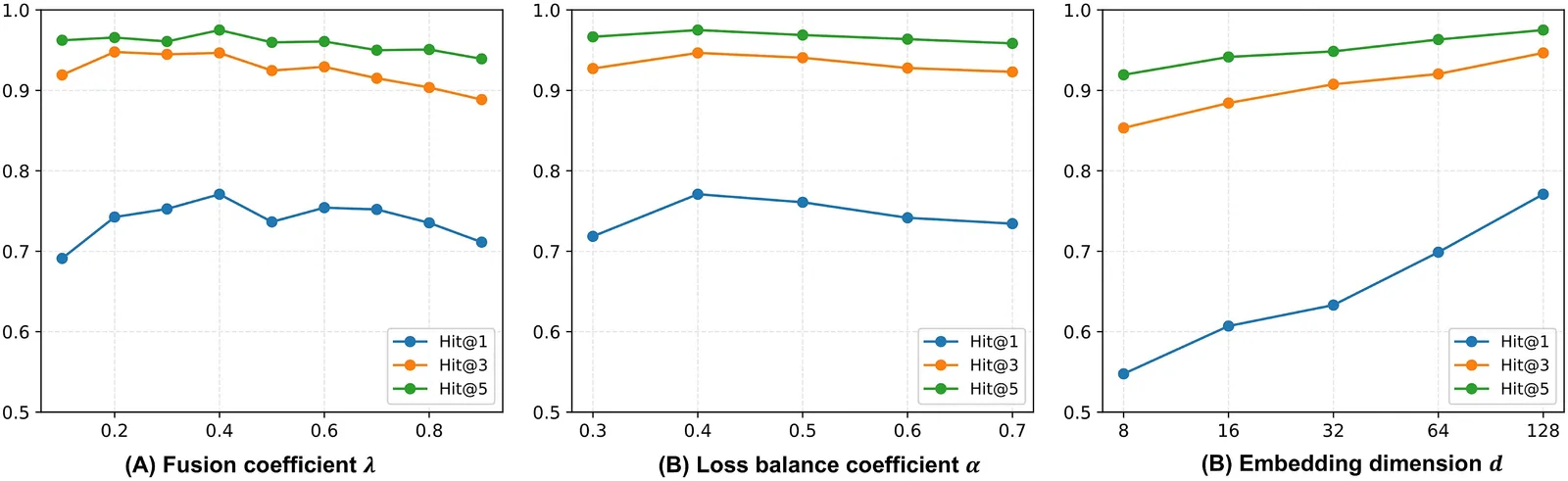
Performance of MTDKF under five-fold cv with different hyper-parameters.

**Analysis of loss balance coefficient *α *:** The coefficient *α * controls the relative weighting of prediction errors from negative and positive samples. As shown in Fig. 5B, the model achieves optimal performance at *α =0.4*. Smaller values lead to insufficient constraint on negative samples, while larger values overemphasize negative samples and degrade performance. These results indicate that an appropriate balance between positive and negative samples is essential for effective training.

**Analysis of embedding dimension *d*:** We further evaluate the impact of embedding dimension *d*, ranging from 8 to 128. As shown in Fig. 5C, the model performance consistently improves with increasing dimensionality. Specifically, when the dimension is small, the model performs relatively poorly and is insufficient to capture node embeddings. As the dimension increases, performance improves significantly, and the model achieves optimal results when *d=128*.

### Identification of miRNAs associated with drug resistance and sensitivity in breast cancer

Breast cancer remains the most frequently diagnosed malignancy among women worldwide. Epidemiological projections estimate that by 2025, *∼ *2.5 million new cases and 700 000 deaths will occur globally each year [30]. Doxorubicin and tamoxifen are widely used anticancer agents in breast cancer treatment, serving as standard therapeutic options for adjuvant therapy, tumor downstaging, and the management of both early-stage and aggressive breast cancer [31]. In this section, we evaluate MTDKF on two breast cancer drugs, doxorubicin and tamoxifen, to validate its capability in identifying miRNAs associated with drug resistance and sensitivity. Specifically, all known MRDR (or MRDS) associations were used to train the model, and predictions were subsequently performed on all unknown miRNA-doxorubicin-breast cancer and miRNA-tamoxifen-breast cancer triplets. To reduce potential prediction bias, the experiments were repeated five times independently. Candidate miRNAs that appeared at least three times among the top 20 ranked predictions were retained as robust predictions. The corresponding results are illustrated in Fig. 6.

**Figure 6:**
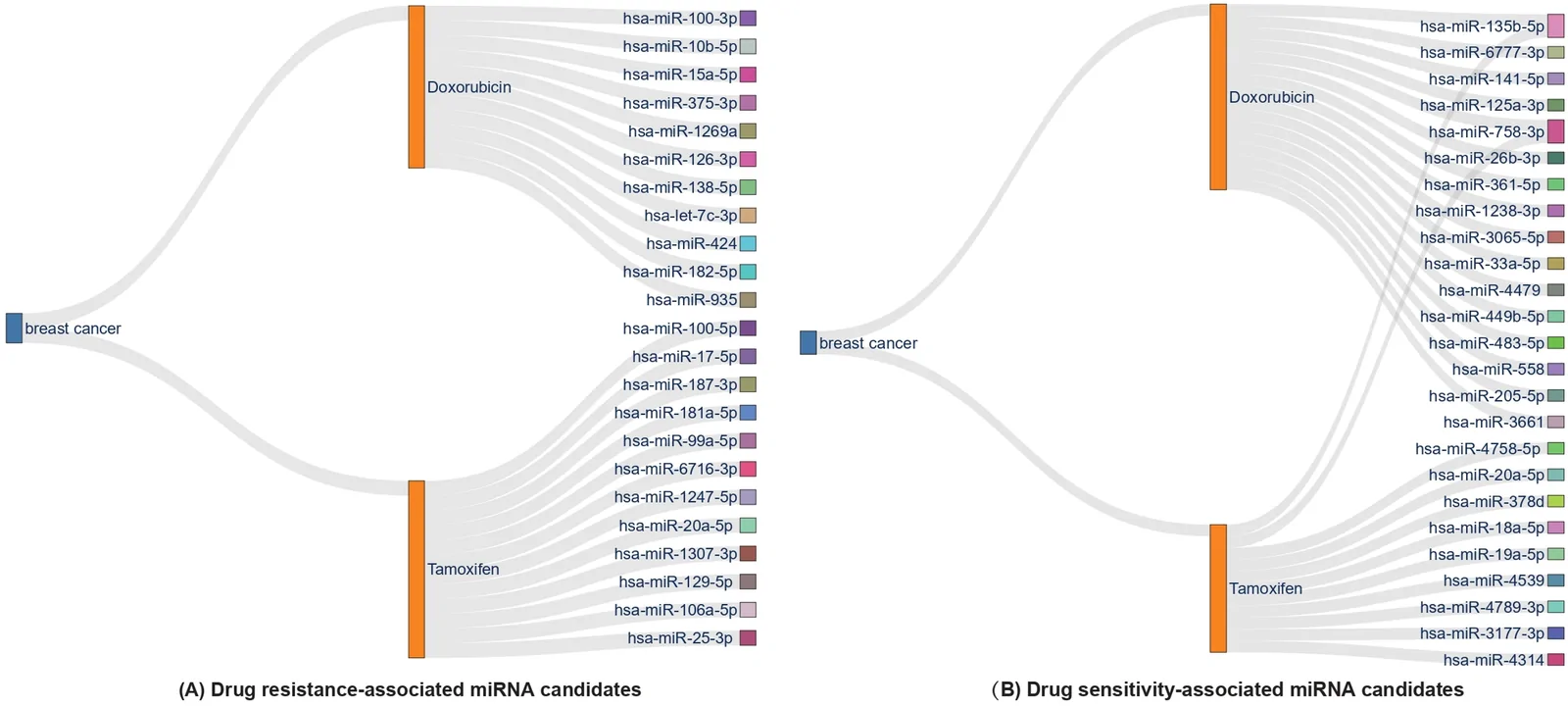
The predicted miRNAs associated with resistance and sensitivity to the breast cancer drugs doxorubicin and tamoxifen by MTDKF.

The results demonstrate that several predicted candidate miRNAs have already been supported by recent experimental studies. For doxorubicin, Mizielska *et al*. [32] evaluated multiple doxorubicin-treated breast cancer cell lines and confirmed that hsa-miR-126-3p is responsive to doxorubicin treatment. In addition, Zhang *et al*. [33] demonstrated in both breast cancer cell lines and clinical breast cancer tissues that miR-135b-5p enhances doxorubicin sensitivity by targeting AGR2. For tamoxifen, Lin *et al*. [34] reported that therapeutic strategies targeting the miR-181a-5p/OSBPL3 axis may provide a promising approach for overcoming tamoxifen resistance in breast cancer. Overall, these findings further support the effectiveness of MTDKF in discovering miRNAs related to drug resistance and sensitivity under disease-specific contexts.

## Conclusion

Unlike existing approaches that focus solely on miRNA–drug pairwise associations, this study incorporates disease context and proposes a novel metapath topology and domain knowledge fusion framework, named MTDKF, for predicting MRDR and MRDS associations. The proposed framework integrates two complementary perspectives: a metapath topology view encoder to capture regulatory patterns, and a domain knowledge view encoder to incorporate intrinsic biological attributes of miRNAs, drugs, and diseases. To support this study, we manually constructed two benchmark datasets covering MRDR and MRDS associations. Experimental results demonstrate that MTDKF consistently outperforms baseline methods across multiple evaluation metrics. Ablation studies further validate the effectiveness of both metapath modeling and domain knowledge integration. In addition, a case study on breast cancer drugs doxorubicin and tamoxifen highlights the potential of the proposed framework in identifying novel miRNAs associated with drug resistance and sensitivity.

In future work, several directions can be explored to further improve MRDR and MRDS association prediction. First, incorporating more diverse biological interaction information, such as gene- and protein-level data, may enrich the underlying network and enhance prediction performance. Second, integrating pretrained biological language models and external knowledge graphs further improve the model’s generalization ability and interpretability.

**Key Points**

- The miRNAs are pivotal regulators of drug resistance and sensitivity in cancer cells, functioning as tumor suppressors or oncogenes that modulate the cellular response to anticancer drugs.
- We propose a novel metapath topology and domain knowledge fusion framework MTDKF that accurately predicts MRDR and MRDS associations.
- The model fuses topology and attribute embedding via weighted fusion mechanism to integrate dual-view information.
- We manually constructed benchmark datasets and experimentally demonstrated that MTDKF outperforms other baseline methods.

## Supplementary Material

Supplementary_file_bbag579

## Data Availability

The source data and code are available at https://github.com/sheng-n/MTDKF.
