# Supplementary material for: Metapath-guided topology and domain knowledge fusion for predicting miRNA–drug–disease triplet resistance and sensitivity associations
Source: Brief Bioinform. 2026 Nov 7;27(6):bbag579. doi: 10.1093/bib/bbag579 (PMC13744293; doi:10.1093/bib/bbag579)
Supplement: Supplementary file 1 [file supplementary_file_bbag579.docx]

**Metapath-guided topology and domain knowledge fusion for predicting miRNA-drug-disease triplet resistance and sensitivity associations**

Nan Sheng^1^, Ling Gao^1^, Xiongfeng Tang^2^, Yunzhi Liu^1^, Lan Huang^1,^*, Yan Wang^1,^*

1. Key Laboratory of Symbol Computation and Knowledge Engineering of Ministry of Education, College of Computer Science and Technology, Jilin University, Changchun 130012, China.

2. The Orthopaedic Medical Center, Second Hospital of Jilin University, Changchun, 130022, China.

Corresponding authors:

**Lan Huang**, huanglan@jlu.edu.cn; **Yan Wang**, E-mail: wy6868@jlu.edu.cn;

**1. Overview of Baseline Methods**

**Random Forest and XGBoost** are classical machine learning methods that use attribute features of the miRNA, drug, and disease nodes as input.

**GCLMTP [1]** is a graph contrastive learning-based representation method that constructs a triplet heterogeneous graph for lncRNA-disease associations, miRNA-disease associations, and lncRNA-miRNA interactions prediction.

**CSGLMD [2]** is a self-supervised graph convolutional network model based on contrastive learning for predicting lncRNA-disease associations, miRNA-disease associations, and lncRNA-miRNA interactions.

**MCHNN [3]** is a hypergraph neural network model based on multi-view contrastive learning for predicting drug-microbe-disease associations.

**HCMGNN** [4] is a graph neural network method based on causal metapath for predicting gene-microbe-disease associations.

**2. Analysis of miRNA sequence length**

Supplementary Figure S1. Distribution of miRNA sequence lengths

**3. Comparison with existing methods on benchmark datasets**

**Supplementary Table S1**. Performance comparison between MTDKF and baseline methods on the MRDR-dataset and MRDS-dataset under 5-fold cv and independent test set.

| MRDR-dataset 5-fold cv | | | | | | | |
| --- | --- | --- | --- | --- | --- | --- | --- |
| **Method** | **Hit@1** | **Hit@3** | **Hit@5** | **NDGG@1** | **NDGG@3** | **NDGG@5** | **MRR** |
| Random Forest | 0.6230 | 0.8001 | 0.8532 | 0.6230 | 0.7293 | 0.7511 | 0.7323 |
| XGBoost | 0.6232 | 0.8390 | 0.8956 | 0.6232 | 0.7514 | 0.7749 | 0.7452 |
| GCLMTP | 0.7377 | 0.8749 | 0.9094 | 0.7377 | 0.8197 | 0.8339 | 0.8176 |
| CSGLMD | 0.7243 | 0.9264 | 0.9660 | 0.7243 | 0.8454 | 0.8617 | 0.8305 |
| MCHNN | 0.7289 | 0.8908 | 0.9272 | 0.7289 | 0.8252 | 0.8403 | 0.8183 |
| HCMGNN | 0.7565 | 0.9243 | 0.9575 | 0.7565 | 0.8562 | 0.8692 | 0.8451 |
| **MTDKF** | **0.7709** | **0.9466** | **0.9750** | **0.7709** | **0.8759** | **0.8877** | **0.8608** |
| MRDR-dataset independent test | | | | | | | |
| Random Forest | 0.6379 | 0.8043 | 0.8582 | 0.6379 | 0.7378 | 0.7596 | 0.7423 |
| XGBoost | 0.6610 | 0.8366 | 0.8967 | 0.6610 | 0.7642 | 0.7887 | 0.7656 |
| GCLMTP | 0.7288 | 0.8967 | 0.9630 | 0.7288 | 0.8299 | 0.8573 | 0.8263 |
| CSGLMD | 0.7365 | 0.9045 | 0.9615 | 0.7365 | 0.8358 | 0.8596 | 0.8301 |
| MCHNN | 0.7319 | 0.8952 | 0.9229 | 0.7319 | 0.8454 | 0.8567 | 0.8437 |
| HCMGNN | 0.7421 | 0.9122 | 0.9516 | 0.7421 | 0.8544 | 0.8771 | 0.8480 |
| **MTDKF** | **0.7673** | **0.9399** | **0.9738** | **0.7673** | **0.8691** | **0.8832** | **0.8556** |
| MRDS-dataset 5-fold cv | | | | | | | |
| Random Forest | 0.5880 | 0.7937 | 0.8705 | 0.5880 | 0.7089 | 0.7406 | 0.7110 |
| XGBoost | 0.5683 | 0.8136 | 0.8915 | 0.5683 | 0.7127 | 0.7453 | 0.7077 |
| GCLMTP | 0.7488 | 0.8737 | 0.9069 | 0.7488 | 0.8239 | 0.8375 | 0.8241 |
| CSGLMD | 0.7330 | 0.9036 | 0.9298 | 0.7330 | 0.8269 | 0.8319 | 0.8331 |
| MCHNN | 0.7341 | 0.8654 | 0.9067 | 0.7341 | 0.8128 | 0.8297 | 0.8128 |
| HCMGNN | 0.7335 | 0.8901 | 0.9340 | 0.7335 | 0.8274 | 0.8456 | 0.8233 |
| **MTDKF** | **0.7542** | **0.9229** | **0.9560** | **0.7542** | **0.8554** | **0.8691** | **0.8447** |
| MRDS-dataset independent test | | | | | | | |
| Random Forest | 0.6327 | 0.8497 | 0.9044 | 0.6327 | 0.7595 | 0.7824 | 0.7519 |
| XGBoost | 0.5811 | 0.8452 | 0.9059 | 0.5811 | 0.7369 | 0.7627 | 0.7248 |
| GCLMTP | 0.7739 | 0.9195 | 0.9529 | 0.7739 | 0.8616 | 0.8754 | 0.8549 |
| CSGLMD | 0.7633 | 0.9454 | 0.9612 | 0.7633 | 0.8726 | 0.8833 | 0.8566 |
| MCHNN | 0.7814 | 0.8877 | 0.9195 | 0.7814 | 0.8457 | 0.8588 | 0.8462 |
| HCMGNN | 0.7739 | 0.9286 | 0.9696 | 0.7739 | 0.8669 | 0.8840 | 0.8584 |
| **MTDKF** | **0.7996** | **0.9650** | **0.9824** | **0.7996** | **0.9002** | **0.9117** | **0.8847** |

**References**

[1]. Sheng N, Wang Y, Huang L et al. Multi-task prediction-based graph contrastive learning for inferring the relationship among lncRNAs, miRNAs and diseases, Briefings in Bioinformatics 2023;24.
[2]. Sheng N, Huang L, Wang Y et al. Contrastive self-supervised graph convolutional network for detecting the relationship among lncRNAs, miRNAs, and diseases. In: 2023 IEEE International Conference on Bioinformatics and Biomedicine (BIBM). 2023, 622-629.
[3]. Liu L, Huang F, Liu X et al. Multi-view contrastive learning hypergraph neural network for drug-microbe-disease association prediction. Proceedings of the Thirty-Second International Joint Conference on Artificial Intelligence 2023;4829-4837.
[4]. Zhang K, Huang F, Liu L et al. Heterogeneous causal metapath graph neural network for gene-microbe-disease association prediction, Proceedings of the Thirty-Third International Joint Conference on Artificial Intelligence 2024;681:6161-6169.
